# Sebacate-Intercalated CaAl-LDH Pigments for Corrosion Protection of Aluminum Alloy

**DOI:** 10.1021/acsomega.5c09717

**Published:** 2025-11-27

**Authors:** Lucas Henrique de Oliveira Souza, Andrea Cristoforetti, Fernando Cotting, Wagner Reis da Costa Campos, Stefano Rossi, Michele Fedel

**Affiliations:** † 54530 Nuclear Technology Development Center, 31270-901 Belo Horizonte, Minas Gerais, Brazil; ‡ Department of Industrial Engineering, 19034 University of Trento, via Sommarive n. 9, 38123 Trento, Italy; § Department of Chemical Engineering, 28114 Federal University of Minas Gerais, 31270-901 Belo Horizonte, Minas Gerais, Brazil

## Abstract

Layered double hydroxides (LDHs) have garnered significant attention in recent years due to their unique structure, which enables the intercalation and controlled release of corrosion inhibitors in response to specific stimuli relevant to corrosion processes. In this study, LDH microparticles intercalated with sebacate (SB) were synthesized to function as a corrosion inhibitor through on-demand release. The microparticles were characterized using scanning electron microscopy, Fourier-transform infrared spectroscopy, X-ray diffraction, and thermogravimetric analysis (TGA). TGA demonstrated that LDH has high thermal stability and that the actual SB content in LDH/SB was estimated to be between 21.2 and 27.6 wt %. The effectiveness of calcium and aluminum-based LDHs intercalated with SB as a corrosion inhibitor was evaluated on aluminum AA5005 substrates, both in bare form and coated with an acrylic layer. Accelerated weathering tests revealing a reduction of approximately 40% of the corroded area and a marked reduction in filiform corrosion during constantly high relative humidity. A dilute electrolyte cyclic fog/dry test also demonstrated a significant reduction in corrosion in dry/wet cyclic aging. The SB species demonstrated potential as a corrosion-inhibiting component under the studied conditions by enhancing the corrosion resistance of the aluminum alloy. This performance was confirmed through electrochemical characterization, including potentiodynamic polarization measurements performed using an Ag/AgCl (3 mol·L^–1^ KCl) reference electrode. The pitting potential (*E*_pit_) values, referred to this reference electrode, showed an increase when SB and LDH–SB were used. The blank sample presented an *E*_pit_ of 0.08 V, while the samples containing SB and LDH–SB presented *E*_pit_ values of 0.50 and 0.36 V, respectively.

## Graphical abstract

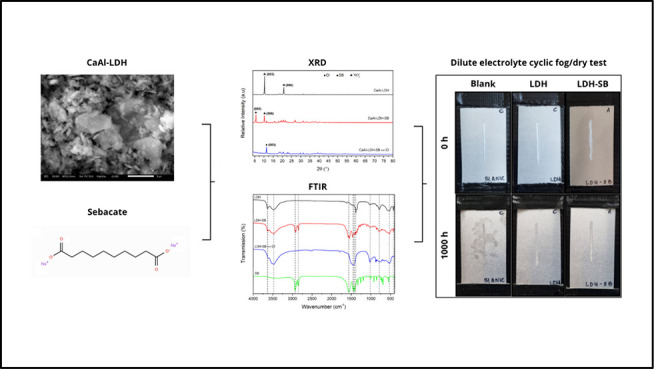

## 1 Introduction

Corrosion remains one of the most significant challenges in the longevity and durability of aluminum alloys, particularly in harsh environmental conditions.^1^ These alloys are widely employed across multiple industries thanks to their favorable strength-to-weight ratio, formability, and aesthetic properties. Among the various aluminum alloys, AA5005 stands out for its balanced combination of mechanical properties and moderate corrosion resistance. It is a nonheat-treatable, high-strength aluminum–magnesium alloy that offers good weldability and an attractive surface finish. Due to these characteristics, AA5005 is extensively used in architectural components, marine environments, transportation sectors, and consumer goods, where both performance and appearance are critical, especially under aggressive environmental conditions. However, their susceptibility to localized corrosion, such as pitting and crevice corrosion, limits their long-term performance without appropriate protective strategies.^2^

Organic coatings represent one of the most widely adopted and effective strategies to enhance the durability of metallic substrates, especially aluminum alloys, in aggressive environments. These coatings act as physical barriers, preventing the diffusion of corrosive agents toward the metal surface. However, their protective performance can degrade over time due to mechanical damage, permeability, or environmental stresses.^3^ To overcome these limitations, modern protective coatings often incorporate active pigments capable of inhibiting corrosion or responding to degradation stimuli.^4^

Among active pigments, a growing interest has emerged toward functional systems capable of providing self-healing or responsive behavior. In this context, layered double hydroxides (LDHs) have attracted attention as smart pigments due to their ability to intercalate corrosion inhibitors and release them on-demand, triggered by the environment stimuli at the corrosion sites.^5,6^ LDHs, also known as hydrotalcite-like compounds, have a unique structure characterized by alternating layers of metal hydroxides and anions. The general formula for LDHs can be represented as [M­(II)_1–*x*_M­(III)_*x*_(OH)_2_]_*x*_+[A^*n*^−]_*x*_/_*n*_·mH_2_O, where M­(II) and M­(III) are divalent and trivalent metal cations, respectively, A^*n* ^–^^ represents the intercalated anions, and m indicates the number of water molecules in the interlayer space.^7^ An example could be the case of calcium–aluminum-based layered double hydroxides (CaAl-LDHs), calcium (Ca^2+^) acts as the divalent metal cation (M­(II)), while aluminum (Al^3+^) serves as the trivalent metal cation (M­(III)).^8^ The typical composition of these pigments involves the intercalation of anions, such as nitrates^9^ or carboxylic acids,^10^ within the hydroxide layers characterized by an excess of positive charge, which enhances their functionality as corrosion inhibitors. The unique arrangement of these metal cations and the intercalated anions contributes to the LDHs’ controlled delivery ability. Recent studies highlighted the potential of LDHs as carriers for corrosion inhibitors, enabling a sustained release mechanism that can significantly improve the protective performance of organic coatings on metals.^8,11–14^ Among various candidates, disodium sebacate (SB) has garnered attention for its efficacy as a corrosion inhibitor due to its ability to form stable complexes with metal ions and its favorable environmental profile.^15^ The incorporation of SB into LDH matrices not only enhances corrosion resistance but also provides an on-demand release mechanism that can respond to localized corrosion events. The on-demand release capability was reported in previous work using the TOC technique.^16,17^ While the SB has been tested for steel substrates both direcly and intercalated, its application to aluminum alloys remains underexplored.^16,17^

This study aims to bridge this gap by synthesizing and characterizing SB-intercalated CaAl-LDHs (LDH-SB) microparticles and evaluating their effectiveness as corrosion inhibitors for AA5005 substrates. Surface protection strategies involving LDH technology have been explored both as conversion coatings and as active pigments embedded within organic matrices, aiming to enhance barrier performance and provide self-healing functionalities in aggressive environments.^18,19^

This research focuses on the impact of the designed LDH-SB system on corrosion protection, particularly its role in mitigating paint delamination and enhancing the overall durability of acrylic coatings when hydrotalcite pigments are incorporated into the coating. By employing various characterization techniques, including scanning electron microscopy (SEM), Fourier-transform infrared spectroscopy (FTIR), X-ray diffraction (XRD), and electrochemical methods, this investigation seeks to provide a comprehensive understanding of the corrosion inhibition mechanisms at play.

The findings of this study are expected to contribute significantly to the field of corrosion science and coatings technology, offering insights into the potential applications of LDH-based smart pigments in protective coatings for aluminum alloys. Ultimately, the goal is to explore the viability of LDH–SB as a promising approach to improving the corrosion resistance of aluminum substrates, thereby supporting the development of more sustainable and efficient protective strategies for various industrial applications.

## 2 Experimental Section

### 2.1 Pigment Synthesis

CaAl-LDHs (named LDH) have been synthesized by dissolving 6.25 g of NaOH and 9.10 g of NaNO_3_ in 44 mL of demineralized water, and under constant nitrogen bubbling, 80 mL of a second solution containing 17.00 g of Ca­(NO_3_)_2_ 4H_2_O and 11.70 g of Al­(NO_3_)_3_ 9H_2_O was added dropwise. The aqueous mixture of the salts (with pH 10.5 at room temperature) was kept in a sealed three-neck flask at 85 °C for 1 h under magnetic stirring conditions. Then, the obtained suspension was collected and centrifuged 3 times at 4500 rpm for 2 min to separate the solid content, which was washed with fresh demineralized water each time. Additionally, the solid powder was washed 3 times using acetone in the same rotary setting. The solid was finally dried in an oven at 40 °C for 24 h and milled to obtain a white powder.^20^ The schematic diagram of the synthesis of CaAl-LDH is shown in Figure 1.

**Figure 1:**
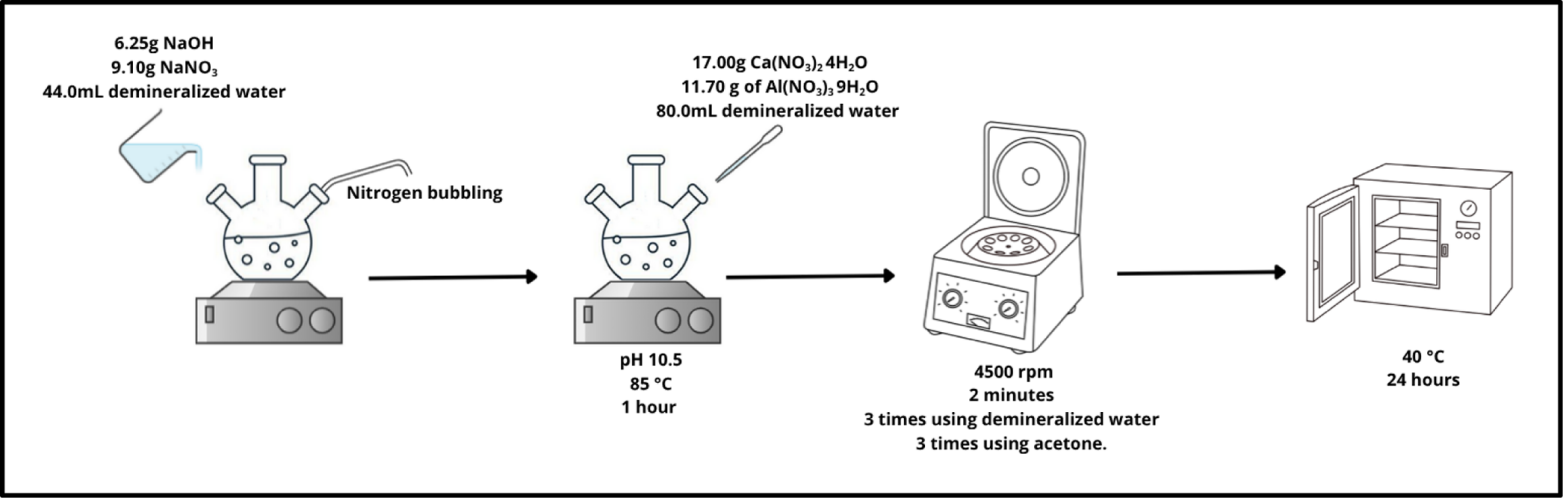
Schematic diagram of CaAl-LDH synthesis.

In the case of the production of the CaAl-LDHs pigments loaded with SB, the synthesis procedure was modified to intercalate and adsorb it in the lamellar structure of the hydrotalcite. In particular, the NaNO_3_ was replaced by 7.00 g of SB (supplied by Merck, Darmstadt, Germany). The rest of the experimental procedure resembled the synthesis described above.

To test the ion exchange performance of synthesized LDH-SB with respect to chloride anions, 3 g/L of pigment was added to a 1 M NaCl solution. Finally, the solution was kept at 25 °C and stirred for 48 h. Then, the powder was washed with the same procedure adopted in the case of the production of the pristine pigment.^5^ The material obtained after the ion exchange test was named LDH-SB-Cl.

### 2.2 Pigment Characterization

To characterize the functional groups, the powder samples were analyzed by FTIR using a Vertex 70v (Bruker, EUA), coupled with ATR platinum diamond (4 cm^–1^ resolution, 128 scans, 4000–400 cm^–1^ wavenumber range).

To analyze the crystal structure of the samples, X-ray diffraction patterns of the pigments were obtained using an X’Pert High Score diffractometer (Rigaku, Japan) with Cu Kα emission source (λ = 1.5046 Å) and the monochromator working at 30 kV and 10 mA. A JSM-IT300 (Jeol, Japan) SEM equipped with an Energy Dispersive X-ray Spectroscopy (EDS) detector was employed to observe the morphology of the LDHs powder structure.

TGA was performed to identify the thermal stability of the samples and to observe the presence of the SB inhibitor in the LDH structures. A DTG-60H analyzer (Shimadzu, Japan) was used to analyze the temperature from 25 to 1000 °C with a heating rate of 10 °C min^–1^. Measurements were performed in a nitrogen atmosphere with a flow rate of 50 mL min^–1^.

To evaluate the behavior of the materials against corrosion and to evaluate the SB inhibition potential, electrochemical impedance spectroscopy (EIS) and potentiodynamic polarization curves (PDP) were performed on AA5005 plates (7.5 cm × 4.0 cm) (composition reported in Table 1) immersed in a 0.01 M NaCl + 0.1 M Na_2_SO_4_ solution at pH 7, to which the LDH-SB and SB powders were added at a concentration of 3.0 g/L. The choice of a mild solution in terms of aggressiveness was made to highlight the effects of the inhibitors and the anionic exchange excess, which in an huge abundance of chlorides could be hidden in the test conditions. In the electrochemical investigation, commercial SB was considered as an independent pigment to compare it to LDH-SB particles to distinguish between the contributing effects of the various compounds. The surface of the AA5005 plates was pretreated as follows: (i) cleaning with acetone under ultrasounds for 6 min; (ii) alkaline etching in 5 wt % NaOH solution at room temperature for 300 s; (iii) dissolution of the aluminum smut layer in 50v/v% of HNO_3_ at room temperature during 5 s. Finally, the samples were thoroughly rinsed with deionized water and dried using compressed clean air. VersaSTAT4 potentiostat galvanostat (Ametek, USA) was used in a three-electrode configuration, where the aluminum alloy panel acted as the working electrode. Platinum and Ag/AgCl/3.5 M KCl electrodes were chosen as the counter and the reference electrodes, respectively. EIS spectra were recorded over the 100 kHz – 0.01 Hz range with a signal amplitude of 10 mV (RMS) and 30 points per decade after 3 h of immersion. EIS results were analyzed in terms of equivalent electric circuits (EEC) using the software Zview2. PDP were collected at a scan rate of 0.16 mV/s polarizing from −0.05 V to +1.50 V vs the open circuit potential after a wait time of 3 h.

**Table 1:** AA5005 Composition

alloying elements (wt %)							
Si	Cr	Cu	Fe	Mn	Mg	Zn	Al
0.3	0.1	0.2	0.7	0.2	0.5–1.1	0.25	balance

### 2.3 Preparation of Acrylic-Coated Aluminum Samples

The protective effectiveness of SB was evaluated on aluminum alloy panels coated with a two-layer system of a bicomponent acrylic-based varnish, supplied by Palini Vernici (Pisogne, BG, Italy). The primer layer was modified by incorporating LDH-SB at a concentration of 1 wt %. LDH-SB was dispersed in the primer with the help of sonication. The topcoat consisted of a paint layer with the same base composition as the primer, applied to prevent the potential leaching of particles toward the outer surface of the coating.

The aluminum substrate was initially cleaned by degreasing with acetone under ultrasonic treatment. Subsequently, the surface was subjected to a pickling process using an aqueous solution containing 5 wt % NaOH, followed by treatment in a solution of 50% v/v HNO_3_ (similar procedure described in section 2.2).

The coating application was performed using an Elcometer 4340 automatic film applicator (Manchester, UK), with a wet film thickness of 100 μm for each layer. Each applied layer was cured at 60 °C for 1 h, resulting in a total dry film thickness of approximately 75 μm. The coated specimens were prepared in accordance with the ASTM D2803 standard.^21^

### 2.4 Characterization and Aging of Acrylic-Coated Aluminum Samples

Three painted samples of each type were aged following the ASTM standard 2803.^21^ A 30 mm long and 1 mm wide scratch was created on each specimen. The coated panels were protected with adhesive tape to avoid early cut-edge failure. Initially, an HCl contamination stage following the DIN EN 3665 standard^22^ was conducted before subjecting the specimens to 1000 h of humidistat aging. During the aging process, the temperature was maintained at a constant 40 °C, and the relative humidity was set at 80% RH.

Similarly, coated panels were also subjected to the dilute electrolyte cyclic fog/dry test in the neutral salt spray chamber following the ASTM G85-A5 standard.^23^ During the dilute electrolyte cyclic fog/dry test, the samples underwent a cyclic fog/dry test using a 0.05 wt % NaCl + 0.35 wt % (NH_4_)_2_SO_2_ solution and heating at 35 °C for 1000 h. The software ImageJ (https://imagej.net/ij/) was used for the determination of the corroded area, a feature monitored during exposure time.

## 3 Results and Discussion

### 3.1 Physicochemical Characterization of the Pigments

#### 3.1.1 Scanning Electron Microscopy

Figure 2 displays the scanning electron microscopy (SEM) images of the synthesized LDH particles without the SB addition. The SEM images primarily reveal plates agglomerates, formed during drying (Figure 2a). However, it is evident that the layered double hydroxides (LDHs) exhibit plates with a hexagonal morphology (Figure 2b,c,d), arranged in multilayered stacks, characteristic of LDH systems, as reported in the literature.^16,20,24–26^ The average plates size was obtained from SEM image analysis using ImageJ software. Examining individual plates, the hexagonal plates exhibit edges measuring approximately 3.09 ± 0.36 μm.

**Figure 2:**
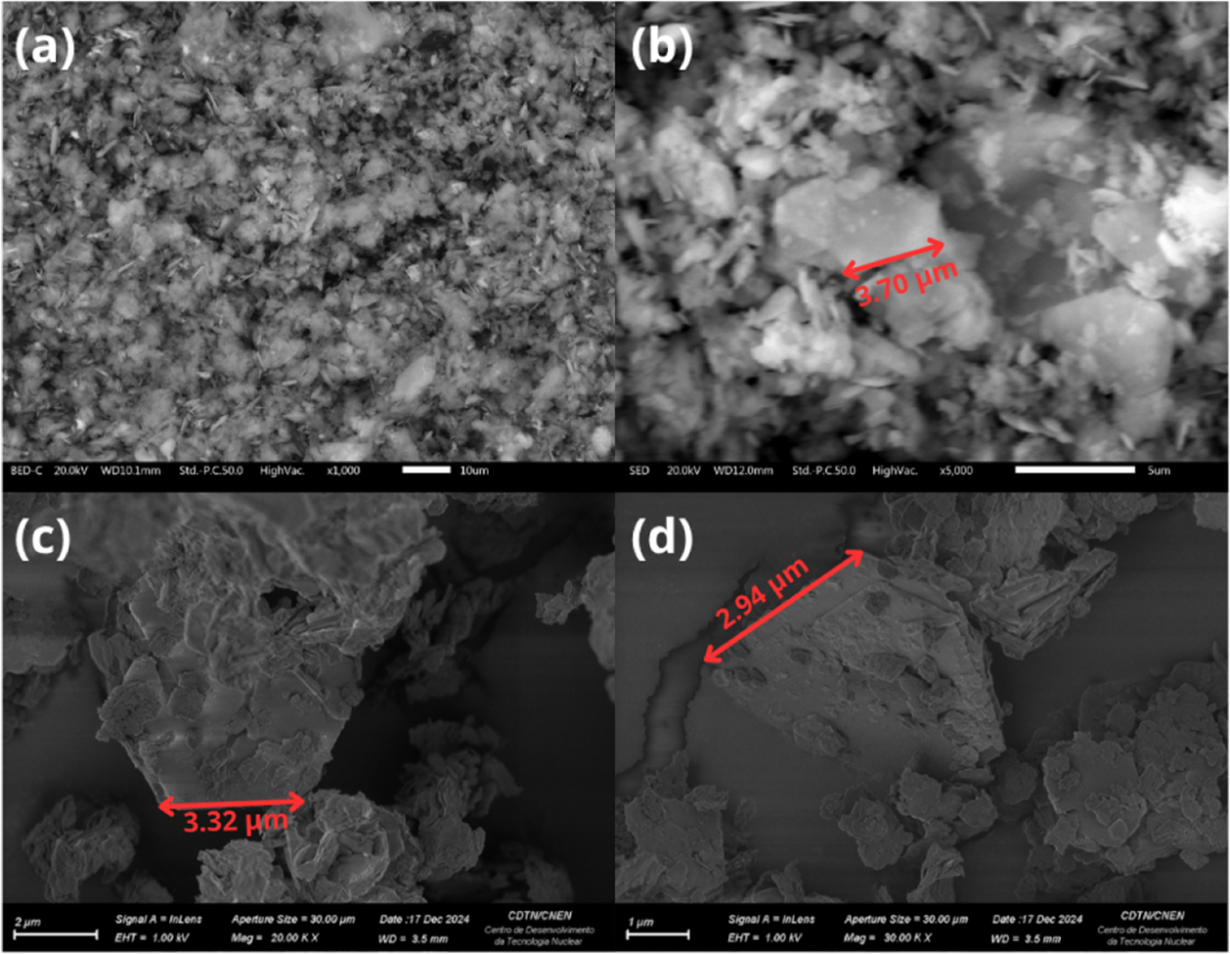
SEM images of LDH particles: (a) 1000×; (b) 5000×; (c) 20000×; (d) 30000×. All images refer to pristine LDH particles without the SB addition.

#### 3.1.2 Powder X-ray Diffraction Analysis

The X-ray diffraction (XRD) patterns of LDH, LDH-SB, and LDH-SB-Cl are presented in Figure 3. For the LDH material, typical LDH peaks were identified at 2θ angles 10.3° and 20.8°, corresponding to the diffraction planes (003) and (006), respectively.^8,10,27^ The application of Bragg’s law (2d sinθ = *n*λ) allows for the determination of the interlayer spacing based on the diffraction peak positions. The type of anion intercalated in the LDH structure can be deduced from the basal spacing (d) value of the (003) peak since the (003) reflection is associated with the intercalation of inhibitors in the interlayer.^10,28^ According to Bragg’s law, the basal spacing (d) value of the (003) peak for LDH was 0.86 nm, which can be attributed to the presence of nitrate ions.^13,29,30^

**Figure 3:**
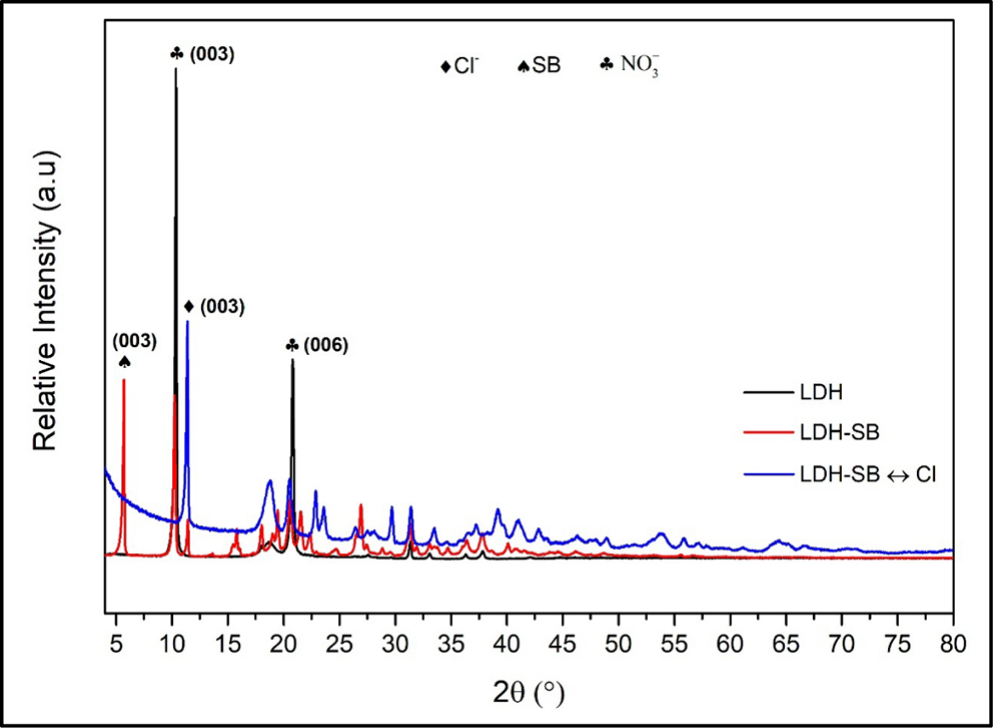
XRD pattern of LDH, LDH-SB, and LDH-SB-Cl.

It can be observed that following the addition of the SB inhibitor to LDH, the typical diffraction peaks of (003) and (003) shifted to lower 2θ angles, presenting 2θ values of 5.67° and 10.18°, respectively.^13^ An increase in the d (003) value for LDH-SB was observed compared to LDH (1.56 nm for LDH-SB vs 0.86 nm for LDH), as expected due to the larger size of the SB anion, indicating the successful intercalation of the SB inhibitor into the interlayer region.^10,17,26^ However, the presence of the characteristic (003) peak located at 10.18° represents the interlayer spacing of the matrix coexisting within the intercalated compounds, indicating an incomplete SB intercalation process.^31^

The partial intercalation of the sebacate anion observed in this study is consistent with the behavior reported for LDH systems containing bulky organic anions. Due to steric hindrance and equilibrium constraints inherent to the intercalation mechanism, complete substitution of nitrate anions by large organic species, such as dicarboxylates, is generally not achievable. Similar results were described by Nguyen et al. (2018),^10^ who reported comparable or even lower intercalation efficiencies for organic inhibitors with similar structures, indicating that our loading values are in line with those reported for analogous systems.

The sample LDH-SB-Cl corresponds to synthesized LDH-SB that was stirred in a 1 M NaCl solution for 48 h. The XRD patterns of LDH-SB-Cl show the same characteristic (003) peak, with a shift of the basal reflection to a higher angle identified at 2θ of 11.41°. The basal spacing (d) value of the (003) peak for LDH-SB after immersion in NaCl was 0.78 nm. This reduced basal spacing indicates that chloride exchange occurred spontaneously with both nitrates and SB initially intercalated in the layers, as intercalated nitrates are replaced by Cl^–^ ions when an exchange reaction is performed in a NaCl solution.^7,27^ The absence of the LDH-SB reflection peak at 5.67° after contact with the saline solution indicates that the stoichiometric excess of chlorides relative to SB and the extended exposure period facilitated a complete exchange process.^10,16^

#### 3.1.3 Fourier Transform Infrared Spectroscopy Analysis

The FTIR spectra LDH, LDH-SB, LDH-SB-Cl, and SB are displayed in Figure 4. In a general way, the three materials presented similar behavior, with bands at 3637 cm^–1^ characteristics for AlO-H^32^ and at 3474 cm^–1^ typical for the stretching vibrations of the O–H functional group and adsorbed water in the interlayer region.^28,30,33^ According to the three materials, the CaAl-LDH structure was recognizable by the peaks at 528 cm^–1^ and 782 cm^–1^ characteristic of Ca–O and Al–O bands, respectively.^8,16^ Typical stretching vibrations of M–O and M–OH were also observed at 1020 cm^–1^.^34^ For CaAl-LDH, the peak at 1350 cm^–1^ belongs to the carbonates, and the peak at 1385 cm^–1^ for the stretching vibrations of the nitrates interlayer.^14,20,26^

**Figure 4:**
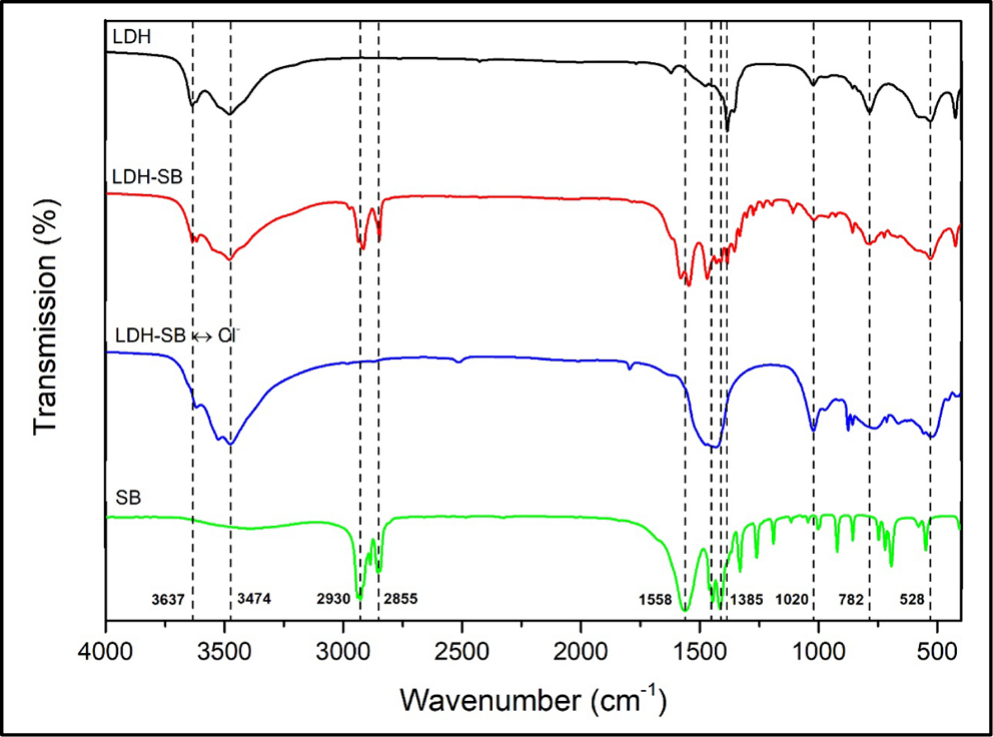
FTIR–ATR spectra of LDH, LDH-SB, LDH-SB-Cl, and SB.

In the spectra of SB, absorption peaks are identified at 2930 cm^–1^ and 2855 cm^–1^, corresponding to the C–H stretching vibrations of SB.^10,17^ The characteristic peaks at 1558 cm^–1^ and 1448 cm^–1^ correspond to asymmetric and symmetric COO^–^ stretching, respectively. The occurrence of two distinct coordination modes of the analogous group in the LDH-SB can be attributed to the presence of two potential metallic elements, calcium (Ca) and aluminum (Al), which are capable of interacting with the carboxylic groups.^10,16,17,35,36^ The bending vibrations at 1420 cm^–1^ correspond to aliphatic chains.^17^

Typical SB bands were identified in the CaAl-LDH-SB spectrum confirming the presence of the SB inhibitor into the CaAl-LDH matter. The absence of typical SB bands in the LDH-SB-Cl spectrum further confirms the complete exchange process, as suggested by the XRD pattern.

#### 3.1.4 Thermogravimetry Analysis

Figure 5 presents the thermogravimetric analysis (TGA/DTG) results, illustrating the thermal decomposition behavior of the LDH, LDH-SB, LDH-SB-Cl, and SB samples as a function of temperature. For the sample containing only the corrosion inhibitor SB, a thermal degradation range is observed between 400 °C and 550 °C, during which a mass loss of approximately 52% occurs. In the case of the LDH sample, the TGA/DTG curve reveals three distinct decomposition stages, resulting in a total mass loss of approximately 42% up to 1000 °C, consistent with values previously reported in the literature.^37^ The first two stages, occurring in the temperature ranges of 48–118 °C and 183–293 °C, are attributed to the release of physically adsorbed water from the surface and the interlayer region, respectively.^38–40^ The third stage corresponds to the decomposition of the LDH structure, involving the release of water resulting from the dehydroxylation of structural OH groups and the loss of hydrogen-bonded water molecules, taking place between 450 °C and 561 °C. The LDH-SB sample exhibits a thermal behavior similar to that of pure LDH, with the addition of a fourth decomposition stage observed in the range of 492 °C to 708 °C. This additional stage is likely related not only to water release but also to the removal of interlayer anions from the hydrotalcite structure.^38,41^ The TGA/DTG curve of the LDH-SB-Cl sample closely resembles that of the pure LDH, indicating the complete release of the corrosion inhibitor SB after exposure of the LDH-SB system to NaCl solution for 48 h.

**Figure 5:**
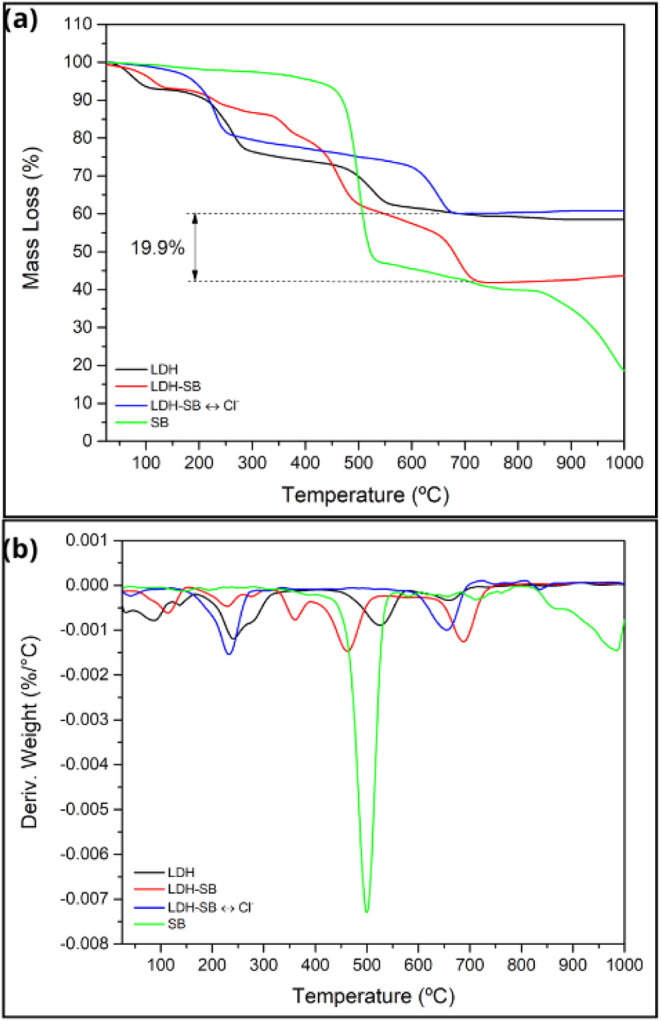
(a) TGA and (b) DTG profiles of LDH, LDH-SB, LDH-SB-Cl, and SB.

TGA analysis of disodium sebacate revealed a major weight loss between 400 and 550 °C, associated with the release of CO_2_, CO, volatile organic compounds, leaving a residual fraction composed of Na_2_CO_3_ and Na_2_O (48 wt %) and char.^35,42,43^ Above 700 °C, further decomposition of Na_2_CO_3_ occurred with additional CO_2_ release and formation of Na_2_O and char.^44^ In contrast, the TGA profile LDH/SB showed no residual sodium-based oxides or carbonates, as the organic anion is retained between the LDH layers in its dissociated form. Consequently, the curve remains stable above 700 °C, indicating the absence of further decomposition. Based on these differences, the residual char content of SB was estimated by considering two limiting cases. Assuming complete conversion to Na_2_CO_3_, the char accounted for 4.9 wt % of the total SB (6.06 wt % when referred solely to the organic anion). Assuming full conversion to Na_2_O, the char content increased to 22.8 wt % (28.0 wt % relative to the organic moiety). From the observed mass loss difference (19.9 wt %) between LDH and LDH/SB samples, and correcting for the expected char residue, the actual SB content in LDH/SB was estimated to range between 21.2 and 27.6 wt %, depending on the assumed nature of the inorganic residue.

The value of SB present in LDH-SB being lower than the values found in the literature^10,16^ was already expected, since the synthesis was carried out for 1 h. Extended synthesis durations do not appear to affect the layered structure, as the XRD pattern remains consistent with that obtained after the 24-h synthesis route. However, intercalation is influenced, likely due to anion exchange, with nitrates being statistically replaced by SB anions in the synthesis environment.

### 3.2 Electrochemical Characterization

Potentiodynamic polarization testing was employed to evaluate the effect of SB and LDH-SB on corrosion inhibition in uncoated aluminum panels immersed in a 0.01 M NaCl + 0.1 M Na_2_SO_4_ solution at pH 7, as illustrated in Figure 6. Polarization curves were recorded after 4 h of immersion in the test solution to allow the system to reach a quasi-steady state. According to the results obtained, the addition of SB to the solution led to a more positive corrosion potential (*E*_corr_), whereas LDH-SB exhibited *E*_corr_ values similar to the reference curve. In all cases, the anodic branches showed a characteristic vertical region, typically associated with materials possessing a passive layer due to the presence of an oxide film.^45^ Moreover, both SB and LDH-SB additions resulted in an increased height of the vertical passive current density plateau, as well as a shift toward higher pitting potential (*E*_pit_) values when compared to the reference sample. The passive behavior observed in the anodic curves, with current densities on the order of 10^–6^ A·cm^–2^, suggests excellent corrosion resistance. The improvement in corrosion resistance can be attributed to the entrapment of aggressive chloride ions, and the controlled release of an optimal amount of inhibitor at defect sites.^8^

**Figure 6:**
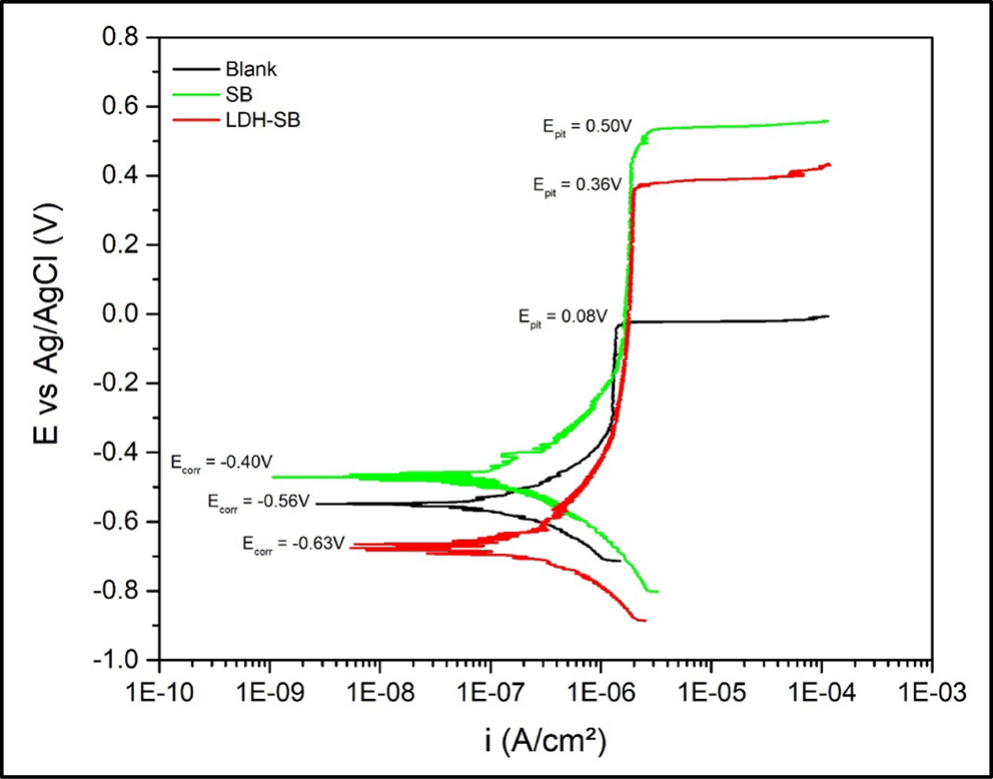
Potentiodynamic polarization curves recorded after 4 h of immersion in a 0.01 M NaCl + 0.1 M Na_2_SO_4_ solution collected on bare steel surfaces where 3 g/L of pigments were added.

Under aggressive polarization conditions and high chloride concentration, the Cl^–^ ion trapping capability of the LDH is not directly observable in the anodic branch of the potentiodynamic curves, as the excess chloride and the continuous potential sweep mask the subtle effects of anion exchange and local chloride depletion. However, under prolonged exposure conditions, with the accumulation of electrolytes at the coating–metal interface, chloride absorption by the LDH becomes more significant, reducing the susceptibility to localized corrosion. This effect is particularly important in painted systems, where the lower availability of chloride near the defect delays the nucleation and propagation of filiform corrosion.^46^ Therefore, while potentiodynamic curves predominantly reflect the anodic behavior under forced polarization, the improvement in corrosion resistance associated with LDH is more clearly evidenced in long-term tests, where electrolyte confinement and film-affected processes occur.

The average values of parameters derived from the polarization curves are presented in Table 2. The superscript letters in Table 2 (a, b, c) refer to the statistical treatment of the data. Results that share the same superscript letter indicate that they are not significantly different from each other and fall within the same standard deviation range. The results demonstrate that both SB and LDH-SB contribute to enhanced corrosion protection of aluminum panels. The replicas of potentiodynamic polarization curves are reported in Figure S1.

**Table 2:** Corrosion Parameters Obtained from Polarization

	*E*_corr_ (V)	*E*_pit_ (V)
Blank	–0.56 ± 0.04^a^	0.08 ± 0.02
SB	–0.40 ± 0.05^b^	0.50 ± 0.07
LDH-SB	–0.63 ± 0.04^a^	0.36 ± 0.02

EIS measurements were carried out under the same experimental conditions as the PDP tests. Figure 7 presents the Bode plots corresponding to the analyzed samples. It was observed that the impedance modulus at low frequency (|*Z*|_0.01 Hz_) exhibited similar values across the three studied conditions.

**Figure 7:**
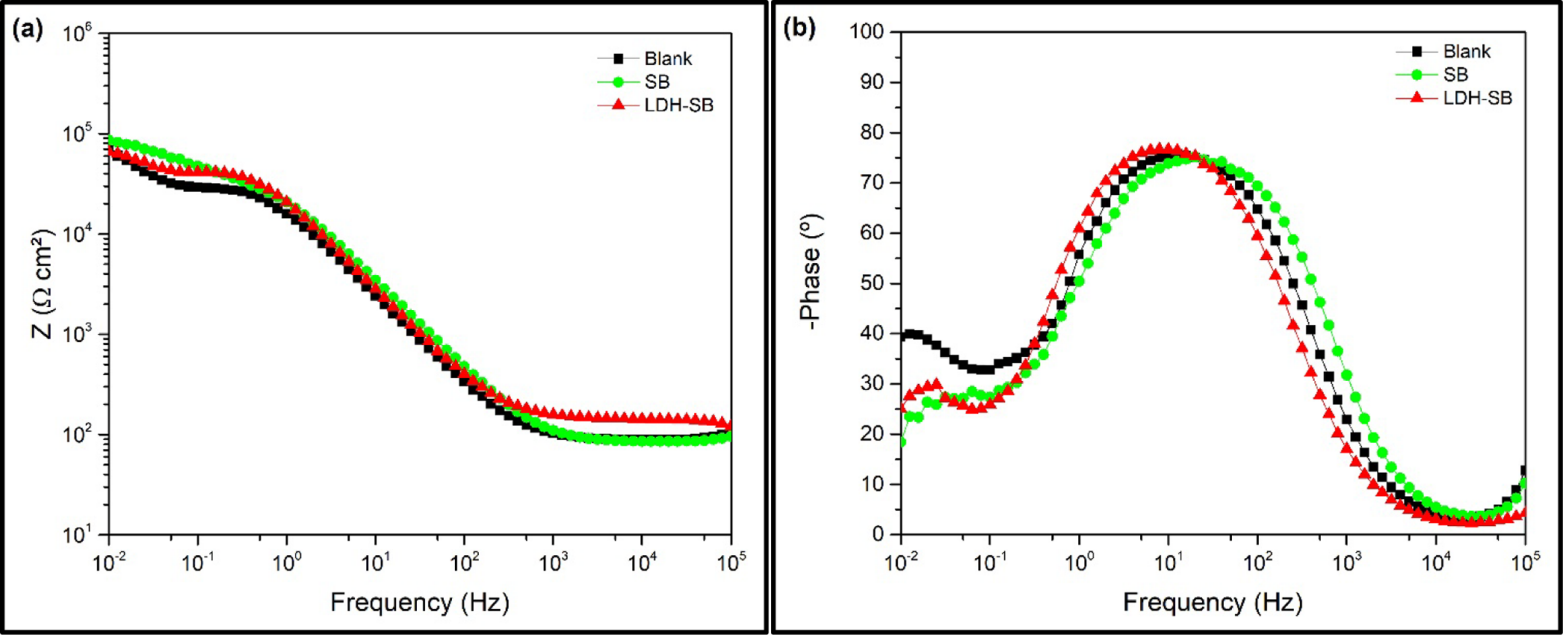
Bode representation of the EIS spectra collected on a bare steel surface in a 0.01 M NaCl + 0.1 M Na_2_SO_4_ solution without and with 3 g/L of pigments in solution after 3 h of immersion. (a) (log |*Z*| vs log *f*) and (b) (phase angle × log *f*).

Figure 8 shows the Nyquist diagrams and the fitting performed corresponding to the analyzed samples. For the quantitative interpretation of the EIS spectra, the equivalent electrical circuit shown in Figure 9 was employed. The R­(QR)­(QR) circuit was used because it provided a significantly better fit to the experimental data. To more accurately account for surface heterogeneities, a constant phase element (CPE) was used in place of an ideal capacitor.^47,48^ In the equivalent circuit, *R*_e_ represents the electrolyte resistance between the reference and working electrodes. The parameters *R*_p_ and *Q*_p_ correspond to the resistance and capacitance associated with the presence of pores. Additionally, *R*_ct_ denotes the charge transfer resistance, while *Q*_dl_ represents the CPE related to the double-layer capacitance.^45^

**Figure 8:**
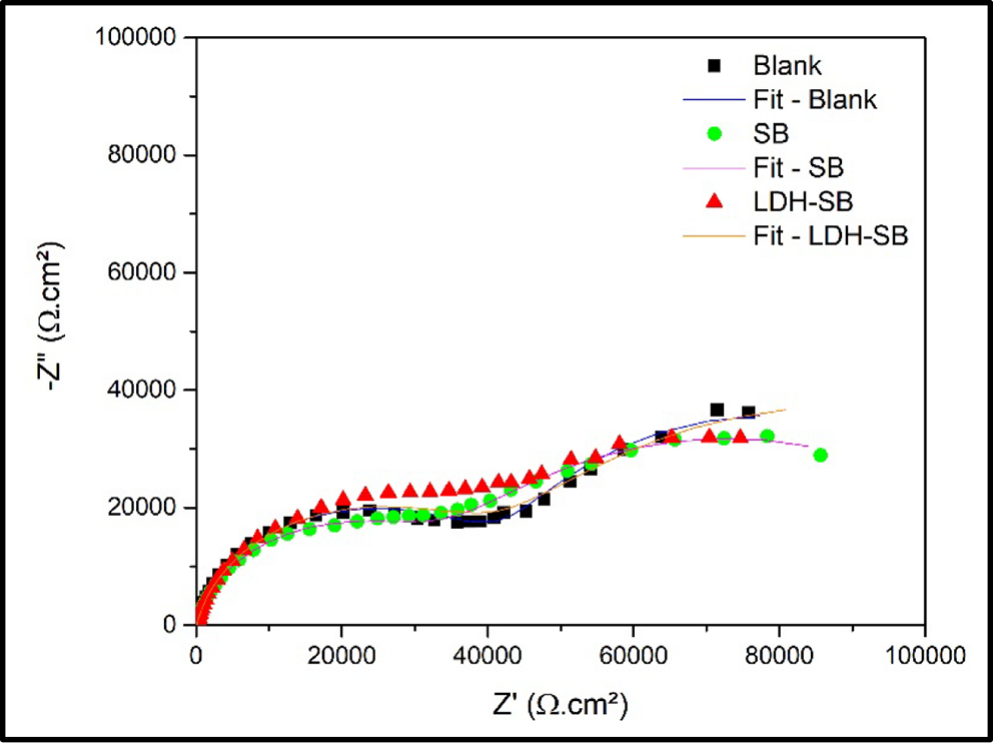
Nyquist representation of the EIS spectra collected on a bare steel surface in a 0.01 M NaCl + 0.1 M Na_2_SO_4_ solution without and with 3 g/L of pigments in solution after 3 h of immersion.

**Figure 9:**
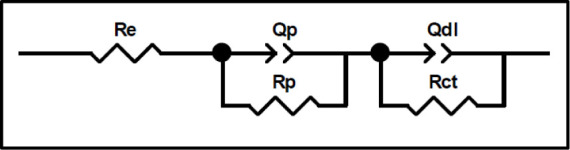
Equivalent circuits used to model the EIS behavior of uncoated aluminum alloys.

The electrochemical impedance spectra exhibited similar behavior across all samples, which may be attributed to the inhibitor release kinetics, suggesting that only a limited amount of SB was released during the immersion period evaluated.

Short-term EIS measurements were purposely conducted to assess the initial electrochemical response and the early activation of the inhibitor in solution. Although long-term immersion EIS could offer complementary insights, such conditions do not accurately reflect real service environments. In practical coated systems, prolonged electrolyte exposure typically represents advanced degradation stages, characterized by coating delamination and corrosion front propagation. Consequently, the long-term inhibition performance is more reliably evaluated through cabinet tests, which simulate cyclic wet/dry conditions and electrolyte accumulation at coating defects.

The replicas of electrochemical impedance spectroscopy are reported in Figure S2. The fitted parameters for each sample are summarized in Table 3.

**Table 3:** Results for EIS Data Fitting^a^

	*R*_e_	error (%)	*Q*_p_	error (%)	*n*_p_	error (%)	*R*_p_	error (%)	*Q*_dl_	error (%)	*n*_dl_	error (%)	*R*_ct_	error (%)	χ^2^
Blank	92.71	0.39	9.62 × 10^–6^	0.89	0.8849	0.52	42274	2.01	1.88 × 10^–4^	3.18	0.9321	0.88	76303	2.44	10^–3^
SB	86.52	0.29	8.52 × 10^–6^	0.58	0.8921	0.47	31892	2.26	7.90 × 10^–5^	4.29	0.7966	1.71	85083	2.84	10^–3^
LDH-SB	104.50	0.81	9.28 × 10^–6^	1.51	0.8932	1.21	38456	3.56	1.06 × 10^–4^	8.74	0.7772	3.49	103870	6.60	10^–3^

a *R*_e_, *R*_p_ and *R*_ct_ (Ω·cm^2^); *Q*_p_ and *Q*_dl_ (*n* F·cm^2^/Sα^–1^) and *N* (α).

### 3.3 Acrylic-Coated Aluminum Steel Corrosion Progression

Aluminum samples with intact coating were evaluated to compare the degradation evolution of coatings immersed in a 3.5 wt % NaCl solution using EIS at regular intervals over a period of 1000 h. The EIS measurements performed on the intact coating during the immersion period did not yield satisfactory results for comparative analysis or degradation monitoring. This is due to the high resistivity of the coating, which resulted in no significant variations between samples or over time. Under all tested conditions, the impedance modulus remained on the order of 10^11^ Ω·cm^2^, indicating a highly effective barrier but one that hinders the extraction of more detailed information through the technique. The EIS spectra on the intact coating are reported in Figure S3.

The damaged coated aluminum samples were evaluated to compare the corrosion evolution in coatings containing LDH and coatings containing LDH-SB with that observed in pure acrylic coatings (Blank). The direct incorporation of free SB into the coating formulation was intentionally avoided because of its high solubility in aqueous environments and poor compatibility with the polymeric matrix. When added as a free species, SB tends to migrate rapidly toward the coating surface or leach out during curing or early immersion stages, leading to inhomogeneous distribution and unreliable evaluation of its long-term inhibitive effect. In contrast, intercalation into the LDH structure ensures a more controlled and sustained release of the inhibitor in response to corrosive stimuli, preventing premature leaching and improving the pigment’s stability and dispersion within the coating.

The tests were conducted under static conditions at 80% relative humidity and a temperature of 40 °C. Figure 10 shows the evolution of the corroded area over 1000 h of aging for the Blank, LDH, and LDH–SB coatings. All systems exhibited a progressive increase in the corroded area with time, as expected under prolonged corrosive exposure. However, the extent of corrosion and the rate of degradation varied significantly among the samples.

**Figure 10:**
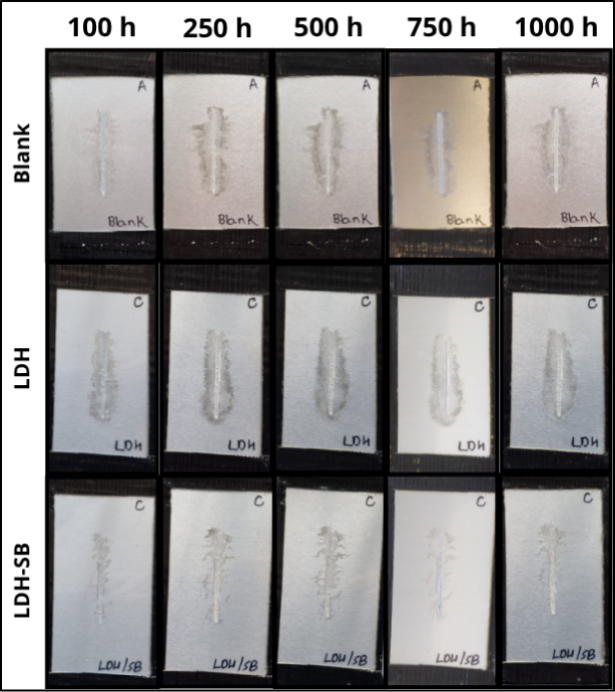
Corrosion status of coated samples scratched during 1000 h of testing at 40 °C in humidostatic conditions (80% RH) after contamination step with hydrochloric acid.

The Blank coating showed a continuous increase in the corroded area, reaching 311.78 ± 36.37 mm^2^ after 1000 h. The LDH coating presented even higher values (415.56 ± 55.37 mm^2^), indicating that LDH alone did not provide effective protection. In contrast, the LDH–SB sample exhibited markedly lower corroded areas throughout the entire exposure period, with 163.08 ± 64.40 mm^2^ after 1000 h. This result clearly demonstrates that the coating incorporating LDH-SB exhibited a reduction in corrosion propagation from artificial scratches, a result attributed to the ability of the SB inhibitor to migrate through the coating matrix and efficiently reach the metallic surface.^15^

In the pure acrylic coating and coatings containing LDH, filiform corrosion constituted the initial event of film delamination, followed by the intensification of corrosion, as evidenced by the formation of white-colored corrosion products and the appearance of large blisters around the initial defect. In contrast, the coating containing LDH-SB exhibited initial-stage filiform corrosion, which remained stable and showed no significant progression after 250 h of exposure. This behavior is attributed to the inhibitory action of SB and the time required for its activation and subsequent migration through the coating matrix to the metal/coating interface.^15^

Figure 11 and Table 4 present the average value of the corroded area expansion around the scratch, calculated from three replicates of each sample type. The superscript letters in Table 4 (a, b, c, etc.) refer to the statistical treatment of the data. Results that share the same superscript letter indicate that they are not significantly different from each other and fall within the same standard deviation range. The inhibitory efficiency of the system was quantified by an average reduction of approximately 40% of the corroded area. The expansion of the affected area was determined using ImageJ software.

**Figure 11:**
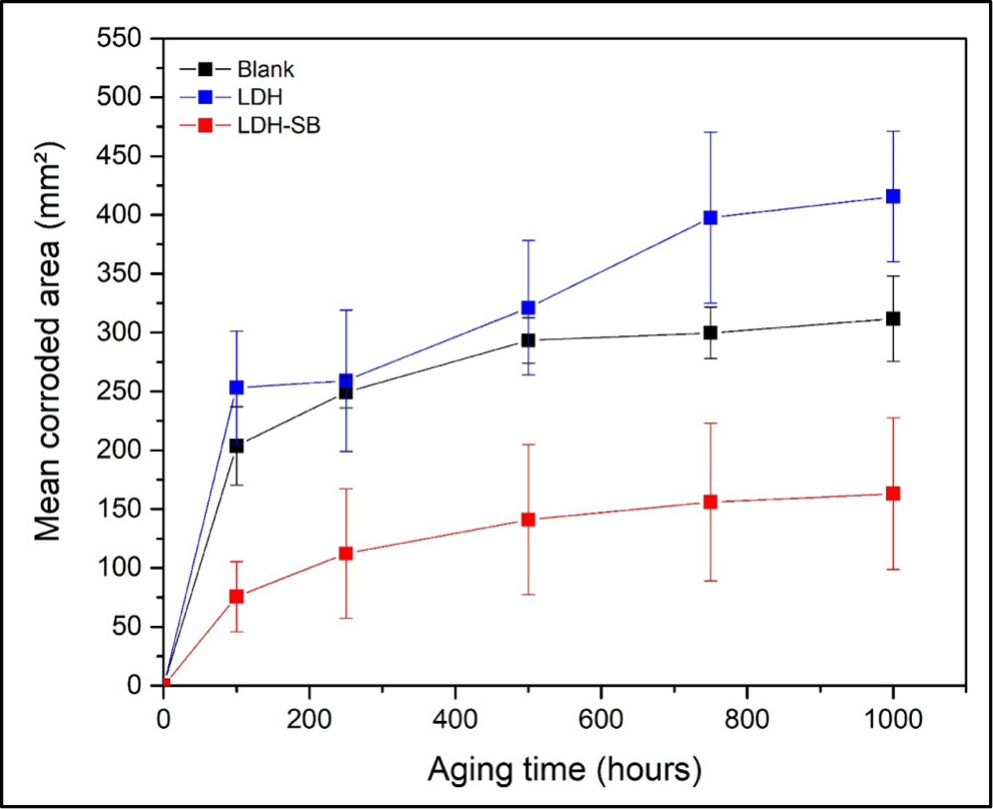
Evolution of the corroded area over 1000 h of aging.

**Table 4:** Data on the Evolution of the Corroded Area over Time During 1000 h of Aging

time (h)	Blank (mm^2^)	LDH (mm^2^)	LDH-SB (mm^2^)
0	0	0	0
100	203.69 ± 33.23^a^	253.17 ± 48.06^a^	75.68 ± 29.72^b^
250	249.09 ± 13.10^c^	259.09 ± 60.03^c^	112.18 ± 54.98^d^
500	293.33 ± 19.30^e^	321.14 ± 57.06^e^	141.02 ± 63.84^f^
750	299.78 ± 21.82^g^	397.68 ± 72.66^h^	155.98 ± 66.90^i^
1000	311.78 ± 36.37^j^	415.56 ± 55,37^k^	163.08 ± 64.40^l^

From a statistical perspective, the error bars in Figure 11 and the standard deviations reported in Table 4 suggest that the differences between LDH–SB and the other systems are statistically significant at nearly all aging times. Although a slight overlap between Blank and LDH error ranges is observed at shorter exposures (up to 500 h), their mean values remain close, indicating statistically comparable behavior within experimental uncertainty. In contrast, LDH–SB values remain consistently outside these ranges, confirming its improved performance. Moreover, the corrosion rate of LDH–SB tends to stabilize after approximately 500 h, suggesting a sustained inhibitor release that effectively delays corrosion propagation.

LDHs significantly influence the nucleation and propagation of FFC on aluminum alloys. They inhibit FFC by sequestering aggressive ions such as chlorides and moderating the pH in the corrosion filament head, which is crucial for controlling the corrosion process.^46,49,50^ The effectiveness of LDH technology is highly dependent on the type of anions exchanged within the structure and in this case, SB further improve the effect. In particular, chloride anions regulate the propagation process of the filaments. These ions are present along the entire length of the filament, even far from the original defect, and their depletion can hinder the compensation of the positive charges generated by the electrochemical activity at the propagating head, ultimately leading to a slowdown in filament growth.^51^

Overall, the incorporation of SB into the LDH structure enhances the coating’s corrosion resistance, leading to a more durable and efficient protective system compared with the Blank and LDH coatings.

### 3.4 Dilute Electrolyte Cyclic Fog/Dry Test

The dilute electrolyte cyclic fog/dry test was conducted to more accurately assess the anticorrosive properties of the samples under investigation. FFC was also observed under the cyclic humidity conditions characteristic of this accelerated aging method.^52^ Figure 12 displays the surface appearance of the samples after 1000 h of continuous exposure. It can be observed that the surfaces of the samples coated with acrylic containing LDH-SB and LDH remained visually intact after the exposure period, with no apparent signs of corrosion. In contrast, the sample coated with pure acrylic (Blank) exhibited significant subfilm corrosion propagation, leading to extensive delamination.

**Figure 12:**
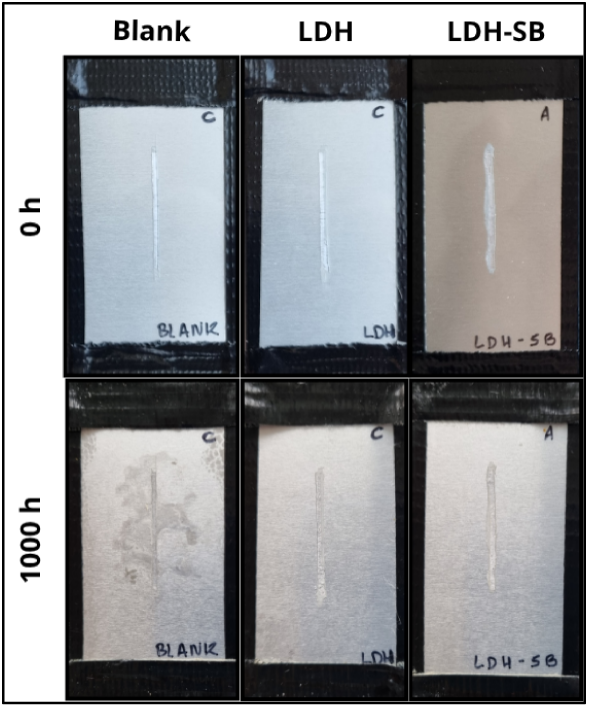
Appearance of the samples after 1000 h of dilute electrolyte cyclic fog/dry test.

Based on the results of the dilute electrolyte cyclic fog/dry test, it can be inferred that the incorporation of LDH-SB and LDH into the coating enhanced its corrosion protection performance, as evidenced by the limited delamination area around the scribed region when compared to the extensive propagation observed in the pure acrylic coating, where FFC developed more intensely. This improved performance is likely attributable to the controlled release of the corrosion inhibitor (SB) in the LDH-SB-containing samples, the chloride-scavenging capability of LDH, and the local alkalization at the coating-metal interface induced by the presence of LDH. This alkalization may inhibit the advancement of corrosion filaments, which typically propagate via anodic undermining mechanisms in acidic environments.

Cyclic conditions significantly enhance the development of FFC by mimicking real-world environmental changes, particularly through alternating wet and dry cycles that facilitate necessary electrochemical reactions. In this context the chloride ions depletion can hinder FFC initiation and propagation also in a cycled aggressive atmosphere, as chlorides contribute to the aggressive environment at the metal/paint interface, promoting anodic dissolution. Without these contaminants, the electrochemical conditions required for FFC are not met, leading to reduced corrosion likelihood.^52^

The differences observed between the humidostatic and cyclic fog/dry tests can be rationalized considering the distinct boundary conditions imposed at the damaged area. Under humidostatic exposure, the absence of continuous external chloride supply causes the electrolyte to remain confined around the scratch, allowing slow evolution of the local chemistry. In this scenario, the film-forming activity of the released SB becomes the dominant protective mechanism, restricting coating delamination and FFC propagation even with limited anion-exchange dynamics. Conversely, in the cyclic fog/dry environment, the recurrent electrolyte renewal introduces a constant flux of aggressive chloride ions, rendering the anion-exchange capability of the LDH phase, particularly chloride uptake and local buffering, an essential factor. However, when chloride ions are abundant and frequently replenished, both LDH and LDH-SB pigments can engage in comparable anion-exchange reactions at the defect, thus reducing the relative benefit offered by SB intercalation. These differences explain why the protective improvement of LDH-SB is more evident under humidostatic conditions, while under cyclic regimes the two systems exhibit similar macroscopic behavior despite potentially distinct underlying mechanisms.

## 4 Conclusions

Based on the results obtained, it can be concluded that LDH microparticles intercalated with disodium sebacate were successfully synthesized through a single-step hydrothermal synthesis process of 1 h. SEM observation revealed that the microparticles exhibit a hexagonal morphology with dimensions around 3.09 ± 0.36 μm. FTIR and XRD analyses confirmed the intercalation of the sebacate anion within the LDH layers, as well as its controlled release after 48 h of immersion in solution. TGA demonstrated that LDH has high thermal stability and that the actual SB content in LDH/SB was estimated to be between 21.2 and 27.6 wt %. Electrochemical assays indicated that SB tends to mitigate corrosion on uncoated aluminum alloys, leading to an increase in pitting potential. Furthermore, accelerated tests on samples coated with an acrylic resin containing the LDH–SB pigment revealed approximately a 40% reduction in delamination at the substrate–coating interface, along with a marked decrease in filiform corrosion and significantly lower corrosion levels in dilute electrolyte cyclic fog/dry test. The LDH-SB system is versatile providing enhanced performance regardless of the environment tested.

## Supplementary Material



## Data Availability

All data supporting the findings of this study are available within the article and its Supporting Information.

## Notes

Replicas of potentiodynamic polarization curves (Figure S1), replicas of electrochemical impedance spectroscopy (Figure S2), and electrochemical impedance spectroscopy of the intact coatings (Figure S3) (PDF)
