# Supplementary material for: Sebacate-Intercalated CaAl-LDH Pigments for Corrosion Protection of Aluminum Alloy
Source: ACS Omega. 2025 Nov 27;10(48):59771–81. doi: 10.1021/acsomega.5c09717 (PMC12771457; doi:10.1021/acsomega.5c09717)
Supplement: Supplementary file 1 [file ao5c09717_si_001.pdf]

Supporting Information

Sebacate intercalated CaAl-LDHs pigments for corrosion protection of aluminum alloy

Lucas Henrique de Oliveira Souza [1,*], Andrea Cristoforetti [2], Fernando Cotting [3], Wagner Reis da Costa Campos [1], Stefano Rossi [2], Michele Fedel [2]

[1] Nuclear Technology Development Center, Belo Horizonte, MG, 31270-901, Brazil.
[2] Department of Industrial Engineering, University of Trento, via Sommarive n. 9, 38123 Trento, Italy.
[3]Department of Chemical Engineering, Federal University of Minas Gerais, Belo Horizonte, MG, 31270-901, Brazil.

***** Correspondance: henrrikelucas@gmail.com;

Lucas Henrique de Oliveira Souza	henrrikelucas@gmail.com
Andrea Cristoforetti	andrea.cristoforetti@unitn.it
Fernando Cotting	fernando@deq.ufmg.br
Wager Reis da Costa Campos	wrcc@cdtn.br
Stefano Rossi	stefano.rossi@unitn.it
Michele Fedel	michele.fedel@unitn.it

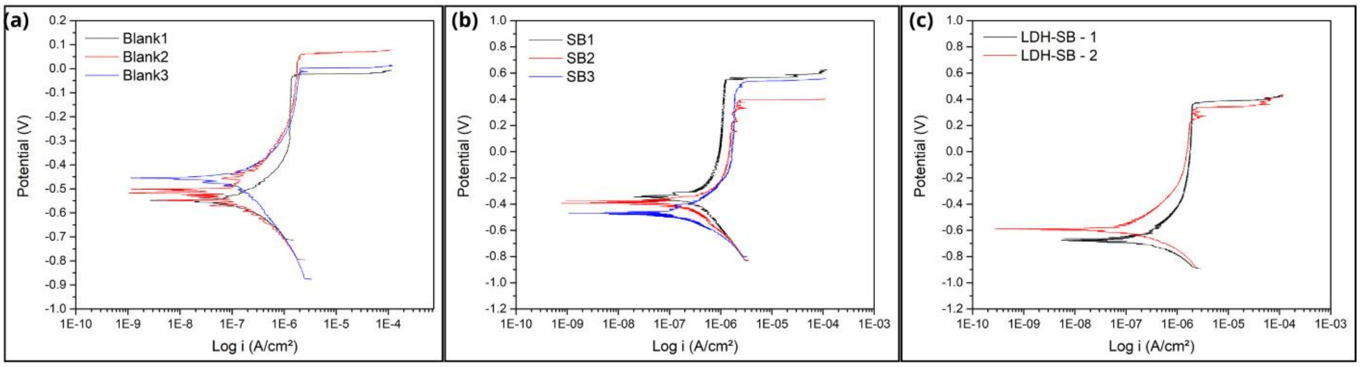

Figure S1. Replicas of potentiodynamic polarization curves. (a) Blank; (b) SB 1%wt; (c) LDH-SB 1%wt.

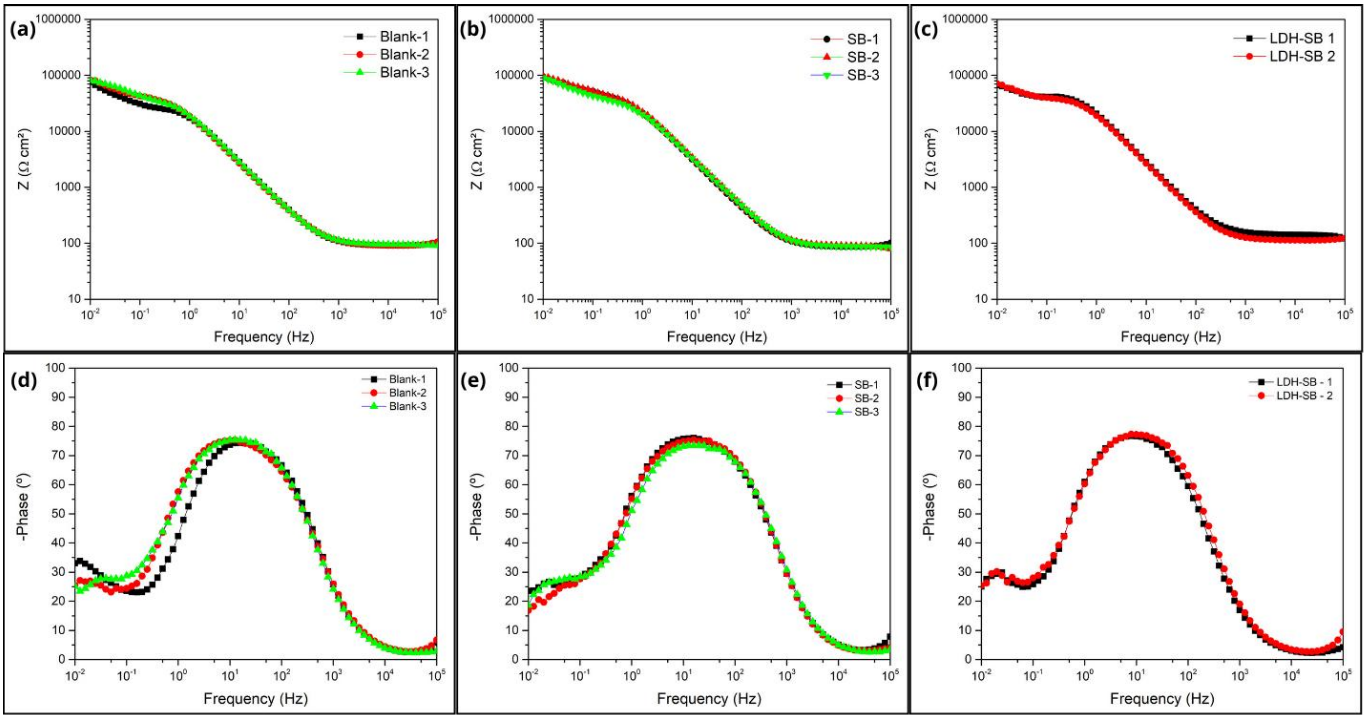

Figure S2. Replicas of electrochemical impedance spectroscopy. (a, d) Blank; (b, e) SB 1%wt; (c, f) LDH-SB 1%wt.

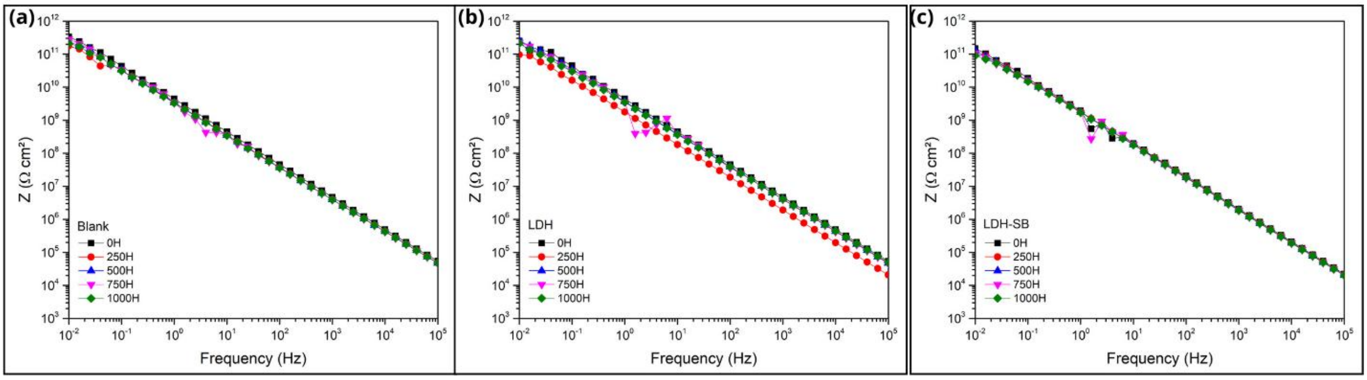

Figure S3. EIS diagrams of the intact coatings during 1000 h in 3.5 wt% NaCl. (a) Blank; (b) LDH 1%wt; (c) LDH-SB 1%wt.
